# Clinical pathways of epileptic seizures and status epilepticus: results from a survey in Italy

**DOI:** 10.1007/s10072-020-04270-3

**Published:** 2020-01-28

**Authors:** Gaetano Zaccara, Giuseppe Citerio, Alfredo Del Gaudio, Monica Ferlisi, Francesco Rocco Pugliese, Danilo Toni

**Affiliations:** 1 Regional Health Agency of Tuscany, via Pietro Dazzi 1, 50141 Florence, Italy; 2 School of Medicine and Surgery, University of Milan-Bicocca, Milan, Italy; 3 Neurointensive Care Unit, San Gerardo Hospital, ASST-Monza, Monza, Italy; 4 DSC Anestesia e Rianimazione 2, IRCCS Casa Sollievo Della Sofferenza, San Giovanni Rotondo, Foggia Italy; 5 UOC Neurologia A, Azienda Ospedaliera Universitaria Integrata, Verona, Italy; 6 Emergency Department, S. Pertini Hospital, Rome, Italy; 7 Department of Human Neurosciences, Sapienza University, Rome, Italy

**Keywords:** Epilepsy, Clinical pathways, Status epilepticus, Acute symptomatic seizure, Unprovoked seizure

## Abstract

**Objective:**

Patients with seizures or status epilepticus (SE) access the hospital through emergency departments and may be admitted into different wards according to the level of care required. Clinicians with different expertise are in charge of taking critical therapeutic decisions. To date, very few studies have investigated the stage at which these patients are referred to neurologists or epileptologists and how guideline recommendations are applied in clinical practice.

**Methods:**

A survey was used to investigate how patients with epileptic seizures or SE are managed in emergency and in subsequent hospital pathways in Italy.

**Results:**

One hundred and seventy-seven physicians (mainly neurologists) from all parts of Italy filled in a questionnaire. Less than half of the participants (35%) answered that, in their hospital, patients with epilepsy were managed by epileptologists. The percentages were lower for patients presenting with acute seizures (21%) or SE (16%). Diagnostic, therapeutic, and assistance pathways (PDTA) for patients presenting with seizure(s) or SE were available for both conditions in about 50% of cases, while, in the rest of the hospitals, participants indicated informal agreements (about 25% of cases) or lack of any agreement (about 25% of cases) between clinicians. Professionals more often involved in PDTA were epileptologists/neurologists, emergency physicians, and intensivists. More than half ot the participants (55%) thought that organizational issues are the most important criticalities for such patients and need to be improved (61%).

**Significance:**

There is a high variability in hospital clinical pathways for epilepsy in Italy.

**Electronic supplementary material:**

The online version of this article (10.1007/s10072-020-04270-3) contains supplementary material, which is available to authorized users.

## Introduction

Epilepsy affects roughly 0.7% of the population [1–3], and is one of the most frequent serious neurologic diseases [4]. In Italy, from official reports, patients with epilepsy are about 500,000 (prevalence rate: 8.5/1000), with an incidence rate of 33.1 new cases each year in every 100,000 residents [5]. For practical therapeutic purposes, patients with epilepsy should be distinguished from those presenting with a first acute symptomatic seizure (seizure occurring in close temporal relationship with an acute brain insult) or those with a first unprovoked seizure (a seizure occurring in the absence of a time-related potentially responsible clinical condition) [6]. While the incidence of acute symptomatic seizures is 29–39/100,000 residents/year [7], the incidence of *isolated* unprovoked seizures has been roughly estimated as 61 per 100,000 person per year, which is consistently higher than the incidence of epilepsy [8].

National Institute for Health and Care Excellence (NICE) epilepsy guidelines (UK) recommend that although initial evaluation of the patient may be undertaken by a primary care physician, all adults and children having a first seizure should be seen as soon as possible by a specialist involved in the management of the epilepsies [9]. However, this is far from being accomplished since such patients may access emergency departments in hospitals where epilepsy specialists are not available and where there are no neurological guards. Therefore, clinicians with a different expertise are often in charge of taking critical decisions regarding their diagnosis and initiation of therapy and very few studies have investigated how early these patients are referred to neurologists or epileptologists and how guideline recommendations are applied in the clinical practice [10].

Status epilepticus (SE), the second most common neurologic emergency after stroke, has an annual incidence of 10–41 cases per 100,000 persons and, in its most severe form (convulsive SE), has a mortality rate that ranges from 7 to nearly 20% [11]. The prognosis of this condition is time-dependent because, if not rapidly controlled, it becomes less sensitive to the treatment and may lead to permanent brain damage and increased mortality [12]. Treatment of SE, as with other neurological emergencies [13, 14], requires a rapid and highly integrated approach involving different professionals. Diagnostic, therapeutic, and assistance pathways (PDTAs) should clearly indicate which professional should take clinical decisions at each specific stage of SE [9]. For purposes of this paper, we considered a PDTA as a formal document approved by all professionals at the hospital or healthcare company level that describes pathways for these patients within the hospital and at discharge.

The aim of this study is to investigate through a survey how patients with epileptic seizures or SE are managed in emergency and subsequent hospital pathways in Italy. Administrative data cannot give us this information because there is no way to discriminate between neurological consultancy and *epileptological* consultancy.

## Material and methods

On October 16, 2018, a meeting was organized to discuss PDTAs of patients with epileptic seizures/seizures in series or SE. This meeting took place simultaneously in six Italian cities (Bari, Bologna, Milano, Napoli, Roma, Torino) which were connected to each other via web conference. Neurologists, epileptologists, emergency room physicians, and intensivists, working in hospitals of different sizes from all parts of Italy and who were actively involved in the treatment of seizures, were invited. Such physicians come from 44 1st level hospitals and from 15 2nd level university hospitals.

Before the meeting, all invited participants were asked to fill in a questionnaire, through an electronic system that guaranteed anonymity. The questionnaire (see supplementary material, table S1) comprised of three groups of questions aimed at describing how patients with seizures or SE are managed in their hospitals. The first group of questions (Qs = 1, 2, 3) sought to find out which professional was involved in the treatment of patients with seizures or epilepsy both in the acute or in the chronic settings. In the second (Qs = 4, 5, 6, 7) and third group of questions (Qs = 8, 9, 10, 11), participants were asked to indicate whether formal or informal clinical pathways of treatment were available for patients presenting with seizure(s) or SE at the emergency department and which professionals were involved in their treatment. We define a *formal PDTA*, as an official document approved by hospital teams and health management of each hospital. An *informal agreement* implies that there was no written document but only verbal agreements between hospital teams involved in the management of these patients. The last two questions were open, and participants were required to indicate what they thought was the most important critical issue in the clinical pathway of such patients (Q = 12) and to make a proposal for improving the indicated critical issue (Q = 13). Participants could skip a specific question if they had no opinion on the issue. Because this was a descriptive survey, all variables were analyzed using only descriptive statistics.

## Results

A total of 177 (75 females; mean age: 49 years) out of the 180 invited participants answered the questionnaire. Among the respondents were 72% neurologists, 16% emergency physicians, 10% intensivists, and 2% other specialists. Based on location, 51%, 19%, and 30% of the participants who answered the questionnaire were from the north, the center, and the south of Italy, respectively.

Answers to questions concerning which professional handles treatment decisions in the acute setting (treatment of seizures or status epilepticus) or is in charge of treatment of people with chronic epilepsy are reported in Table 1.

**Table 1:** Professional involved in the treatment of patients with seizure(s) and epilepsy based on participants’ responses

	In your hospital which physician		
	Is in charge of treatment of patients with chronic epilepsy? (*n* = 166)*	Takes treatment decisions for patients with seizures (*n* = 137)*	Takes treatment decisions for patients with status epilepticus? (*n* = 157)*
Neurologists	85 (51%)	72 (52%)	56 (35%)
Epileptologists	59 (35%)	29 (21%)	26 (16%)
Usually epileptologists but sometimes neurologists	NA	33 (24%)	25 (16%)
Usually neurologists or epileptologists and, sometimes, emergency physicians or intensivists	NA	–	39 (25%)
Usually neurologists or epileptologists and, sometimes, specialists in internal medicine, emergency physicians and intensivists	NA	–	6 (3.8%)
Other specialists	22 (13.0%)	3 (2.2%)	5 (3 > 2%)

*Total number of participants who answered each specific question. *NA*, not applicable. These answers could not be selected after the first question

### PDTA of patients presenting at the hospital because of a single or repeated seizure

Eighty-seven participants (49%) answered that there was a formal PDTA in their hospital. Within the group of those without a formal PDTA (*n* = 90), 53 participants (59%) indicated they had informal agreements and 37 participants (41%) confirmed the lack of any agreement between hospital teams involved in the management of these patients. Table 2 shows the professionals included in formal PDTA or in informal agreements for the treatment of such patients.

**Table 2:** Professionals included in the PDTA for patients presenting at emergency services with seizure(s), based on participants’ responses

	Which professionals are included in the PDTA for patients presenting at the hospital with single or repeated seizures?	
	Professionals included in a formal PDTA (*n* = 87)*	Professionals involved in informal agreements (*n* = 53)*
Epileptologists/neurologists, emergency physicians	22 (25%)	6 (11%)
Epileptologists/neurologists, emergency physicians, intensivists	49 (56%)	33 (62%)
Epileptologists/neurologists, emergency physicians, intensivists, specialist in internal medicine	15 (17%)	14 (26%)
Other specialists	1 (1.2%)	–

*Total number of participants who answered each specific question

### PDTA of patients presenting at the hospital because of SE

Eighty-seven participants (49%) answered that in their hospital there was a formal clinical pathway. Within the group without a formal pathway (*n* = 90), 46 participants (51%) affirmed that there were informal agreements, and 44 participants (49%) stated a lack of any kind of agreement between clinicians involved in the treatment of this condition. Table 3 shows the professionals involved in the treatment of patients with SE.

**Table 3:** Professionals included in the PDTA for patients with status epilepticus, according to the opinion of participants

	Which professionals are included in the PDTA of patients presenting at the hospital with status epilepticus?	
	Professionals included in a formal PDTA (*n* = 87)*	Professionals involved in informal agreements (*n* = 46)*
Epileptologists/neurologists, emergency physicians	23 (26%)	11 (23.9%)
Epileptologists/neurologists, emergency physicians, intensivists	48 (55%)	26 (56.5%)
Epileptologists/neurologists, emergency physicians, intensivists, specialist in internal medicine	16 (18%)	8 (17.4%)
Other specialists	–	1 (2.17%)

*Total number of participants who answered each specific question

The two open questions on (1) what the participants thought was the most important critical issue for the treatment of patients with seizures or SE and (2) their proposals to improve the above reported critical issues were answered by 80 and 74 participants, respectively. Interestingly, more than half (*n* = 44; 55%) answered that the most important critical aspects concerned organizational issues while only 19 (23.5%) focused on staff shortage (in particular, a lack of neurophysiopathology technicians) or facility shortages. Similarly, proposals for improvement were focused on organizational issues (*n* = 45; 61%), and only a minority of cases suggested a need for more facilities (*n* = 17; 23%) which in the majority of cases was the availability of EEG in the emergency. From inspection of answers, there were minor differences in participants’ responses based on location. Organizational issues were more important for participants from the north of Italy while shortage or lack of facilities were more important to those from the south.

## Discussion

This survey was filled in by physicians, mainly neurologists, selected from hospitals of different sizes from all parts of Italy. Although the sample for this study is relatively small, all participants were involved in the management of these patients; therefore, we think that this survey gives a relatively precise picture of how patients with seizures, epilepsy, or SE are managed in Italy. However, a limitation of this survey is that only a small number of intensivists and a relatively low number of emergency physicians were included. A further limitation is that since the questions required simple answers, the complexity of the clinical decision process was inevitably lost.

We found that in the vast majority of hospitals, treatment of chronic epilepsy and acute seizure(s) or SE is managed mainly by general neurologists. Interestingly, in 10% of cases, specialists in internal medicine are also in charge of treatment of patients with chronic epilepsy. Only 35% of respondents stated that neurologists with special expertise in epilepsy (epileptologists) are those most often assigned to treat patients with epilepsy in their hospital. In addition, such professionals take treatment decisions for patients presenting with acute seizures in only a quarter of cases and are involved in the treatment of SE in less than a fifth of cases.

In the literature, there are only a few other examples of how epilepsy patients are managed in the hospital. In an audit performed in the UK, it was observed that of about 1000 patients attending at the hospital because of a first seizure, only 55% were referred to a neurologist or epilepsy specialist [10].

A second finding is that a PDTA does exist for patients with seizure(s) or SE only in about half of the hospitals, while there are no specific procedures or there are informal agreements between clinicians in the rest of the hospitals. In addition, it can be noted that both in the case of PDTA or in the case of informal agreements, the professionals included in the pathway vary greatly. It can be speculated that either there is no agreement on which professionals should be involved in treatment of such conditions and/or these heterogeneities reflect contingent local situations. Multi-professional teams in charge of taking treatment decisions in such conditions may be different, apparently without reasons dictated by the clinical condition of the patient.

All these findings point to the equity of access to healthcare [15]. Keeping sustainability in mind, the most appropriate specialist for the treatment of each specific stage of these conditions should be properly identified. For example, efforts should be made to identify the subpopulation of patients who need an early evaluation by epileptologists and, in such cases, pathways should allow early referral to such specialists.

SE is one of the most frightening medical emergencies and requires expertise, particularly in its refractory form [12]. Unfortunately, only 20% of respondents stated that treatment of SE is managed by epileptologists. Earlier phases of treatment for these patients are often overseen by neurologists without particular expertise in epilepsy and also by emergency physicians, intensivists, and specialists in internal medicine.

Interestingly, the answers to the open questions on the most important critical issue in the clinical pathway of these patients and on how to improve assistance show that there is a perception that criticalities are due mainly to organizational gaps and lack of appropriate PDTAs. Only a few participants thought there was a need for more professionals or facilities.

In conclusion, patients who present at the hospital with repeated seizures or SE need to be managed by a network of professionals and services. For optimal treatment, these professionals should interact with each other and an appropriate sequence of interventions should be laid out in a structured PDTA. Hospitals should consider that imprecise diagnosis and inadequate treatment can have serious clinical consequences and increase costs not only in the short term but also in the long term. For example, long-term toxicity and drug–drug interactions which affect prognosis of such patients may be strongly influenced by the choice of antiepileptic drug.

The multidisciplinary team that should take care of these patients should be clearly identified and should be identical in all hospitals. In those hospitals where all facilities are not available, efficient networks between hospitals should be organized.

## Electronic supplementary material

ESM 1 (DOCX 16.5 kb)
