# Supplementary material for: Clinical pathways of epileptic seizures and status epilepticus: results from a survey in Italy
Source: Neurol Sci. 2020 Jan 28;41(6):1571–5. doi: 10.1007/s10072-020-04270-3 (PMC7275936; doi:10.1007/s10072-020-04270-3)
Supplement: Supplementary file 1 — (DOCX 16.5 kb) [file 10072_2020_4270_MOESM1_ESM.docx]

Supplementary material

**Table S1**

**Questionnaire**

| Number | Questions | Choose the most appropriate answer |
| --- | --- | --- |
| 1 | In your hospital patients with epilepsy are treated by: | 1. Neurologists 2. Epileptologists 3. Other specialists |
| 2 | In your hospital treatment decision on seizures is taken by: | 1. Neurologists 2. Epileptologists 3. Usually by epileptologists but sometimes by neurologists 4. Usually by neurologists but sometimes by emergency physicians or intensivists 5. Usually by neurologists but sometimes by specialists in internal medicine 6. Other specialists |
| 3 | In your hospital treatment decisions on the status of epilepticus are taken by: | 1. Neurologists 2. Epileptologists 3. Usually by epileptologists but sometimes by neurologists 4. Usually by neurologists but sometimes by emergency physicians 5. Usually by neurologists but sometimes by specialists in internal medicine 6. Other specialists |
| 4 | Is there in your hospital a structured clinical pathway for the multidisciplinary team, which takes care of patients presenting in the emergency department with a seizure or repeated seizures? | 1. Yes  2. NO |
| 5 | If the answer is yes, mark clinicians involved in the multidisciplinary team | 1. Epileptologists/neurologists, emergency physicians 2. Epileptologits/neurologists, emergency physicians, intensivists 3. Epileptologits/neurologists, emergency physicians, specialists in internal medicine, intensivists 4. Other |
| 6 | If the answer is no, there is at least ~~an~~ a usual relationship with a verbal discussion between different specialist? | 1. Yes  2. NO |
| 7 | If the answer is yes, mark clinicians usually involved in the discussion | 1. Epileptologists, neurologists, emergency physicians 2. Epileptologists/neurologists, emergency physicians, intensivists 3. Epileptologists/neurologists, emergency physicians, specialists in internal medicine, intensivists 4. Other |
| 8 | Is there in your hospital a structured clinical pathway for the multidisciplinary team, which takes care of patients presenting in the emergency department with status epilepticus? | 1. Yes  2. NO |
| 9 | If the answer is yes, mark clinicians involved in the multidisciplinary team | 1. Epileptologists, neurologists, emergency physicians  2. Epileptolologits/neurologists, emergency physicians, intensivists  3. Epileptologists/neurologists, emergency physicians, specialists in internal medicine, intensivists  4. Epileptologists/neurologists, emergency physicians, specialists in internal medicine, intensivists  5. Other |
| 10 | If the answer is no, there is at least ~~an~~ a usual relationship with verbal discussion between different specialist? | 1. Yes 2. NO |
| 11 | If the answer is yes, mark clinicians usually involved in the discussion | 1. Epileptologists/neurologists, emergency physicians  2. Epileptologists/neurologists, emergency physicians, intensivists  3. Epileptologists/neurologists, emergency physicians, specialists in internal medicine, intensivists  5. Other |
| 12 | In your hospital which is the most important critical issue in the clinical pathway of patients with seizures? | Express your personal opinion in a few lines |
| 13 | Which is your proposal to improve the above reported critical issues? | Express your personal opinion in a few lines |
